# Supplementary material for: Neural representations of honesty predict future trust behavior
Source: Nat Commun. 2019 Nov 15;10:5184. doi: 10.1038/s41467-019-13261-8 (PMC6858375; doi:10.1038/s41467-019-13261-8)
Supplement: Supplementary file 1 — Supplementary Information [file 41467_2019_13261_MOESM1_ESM.pdf]

Supplementary Information

for

Neural representations of honesty predict future trust behavior

Bellucci et al.

Supplementary Figures

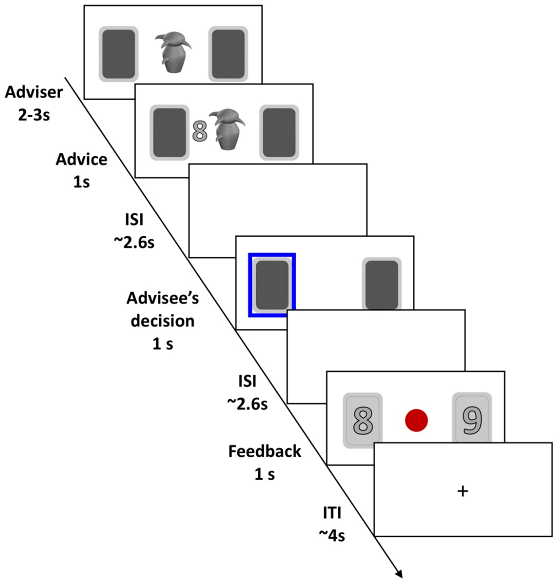

Supplementary Figure 1. Timeline of the Take Advice Game.

Timeline of the Take Advice Game (TAG) in the MRI scanner. Before the task, participants had to choose an avatar that represented their identity in the game. They were told that the advisers did the same. Through these avatars, it was assured on the one hand that participants knew in each trial who advised them and, on the other, anonymity was guaranteed to each participant. In the adviser presentation phase, participants were told that they would see the adviser they were matched with in that trial. In this phase, the adviser was given information about one of the two cards that he or she could communicate to the participants. Thus, in the adviser phase, participants needed to wait that the adviser sends her/his advice. To introduce human-like decisional variability in the communication of the advice, the advice was randomly presented between 2 and 3 seconds after adviser presentation. The advice (presentation time: 1s) was a number between 1 and 9 (expect for 5) either next to the right or the left card. After a variable ISI (range: 2-8s, mean: 2.6s), the two cards were presented one more time and participants were prompted to pick one of the two cards (1s). After another variable ISI, feedback was presented for 1s, revealing the numbers on the cards, based on which participants could judge the honesty of the adviser (social information), and a red or green circle between the cards, representing the participant's performance (nonsocial information about own payoffs). Finally, an ITI (range: 2-8s, mean: 4s) showing a fixation cross was presented at the end of the trial.
ISI, interstimulus interval; ITI, intertrial interval.

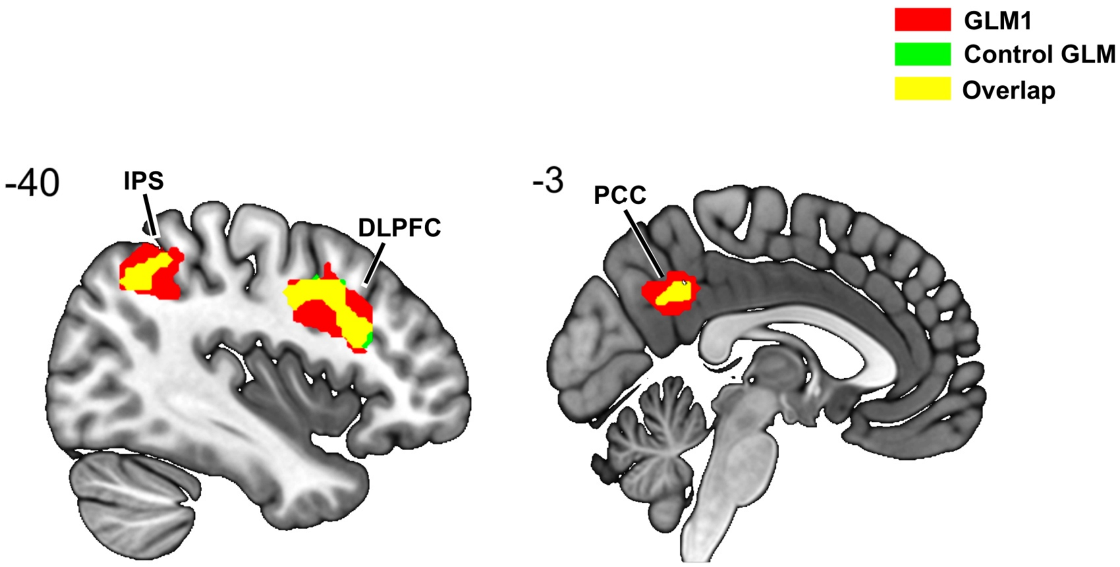

Supplementary Figure 2. Control GLM analysis.

Comparison of multivariate results based on GLM1 coding for honesty and dishonesty (red), and on the control GLM that further controlled for risk and congruency effects (green). The two GLMs yielded similar results. In yellow are the overlaps depicted.
GLM, general linear model; IPS, intraparietal sulcus; DLPFC, dorsolateral prefrontal cortex; PCC, posterior cingulate cortex

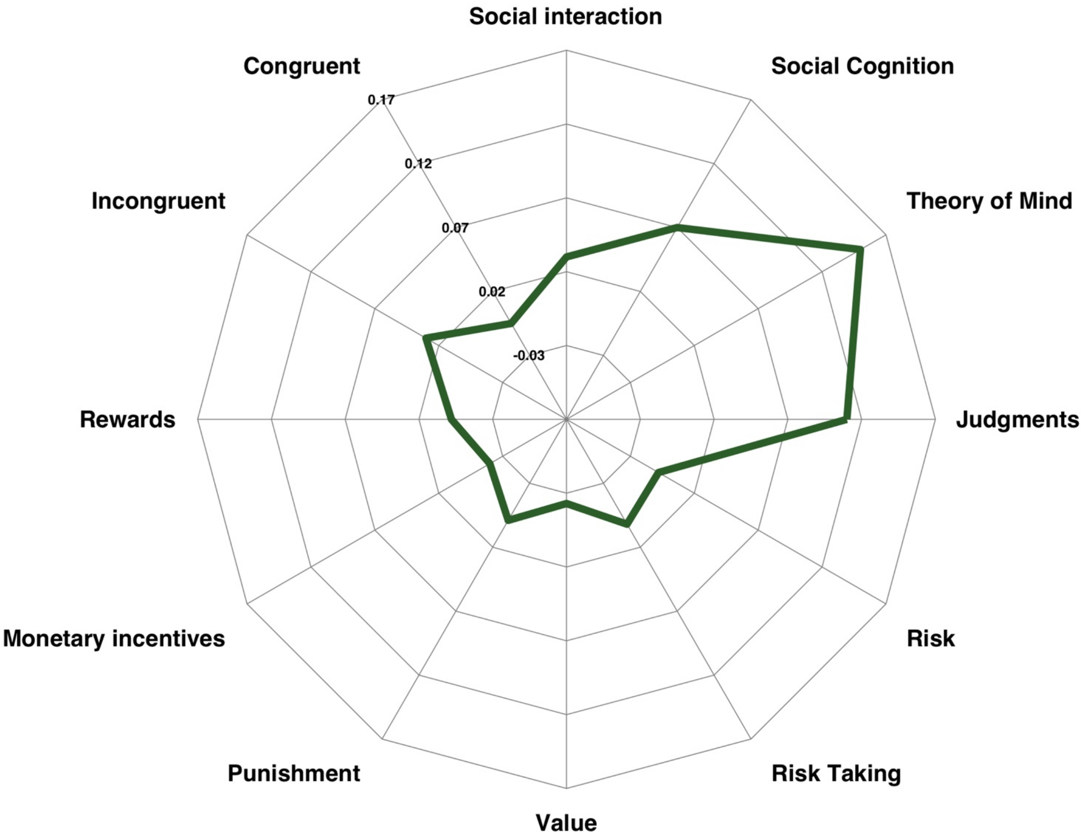

Supplementary Figure 3. Meta-analytic functional decoding analysis.

To test the functional specificity of the trustworthiness decoding network, we performed a meta-analytic functional decoding analysis. Using the Neurosynth database, we evaluated the representational similarity between the neural patterns of our trustworthiness map and meta-analytic neural patterns associated with specific terms in the fMRI literature. This way, it was possible to characterize the functional role of our neural patterns by a quantitative comparison with previously observed neural patterns associated with certain cognitive functions. We selected twelve different terms in the social, value, risk and congruency domain. Results show that the neural signatures of the trustworthiness decoding network reveal stronger similarity with neural patterns associated with mentalizing, judgments, social cognition and social interactions than with other cognitive functions. Values on the spider plot (-0.03 — 0.17) represent Pearson's correlation coefficients.

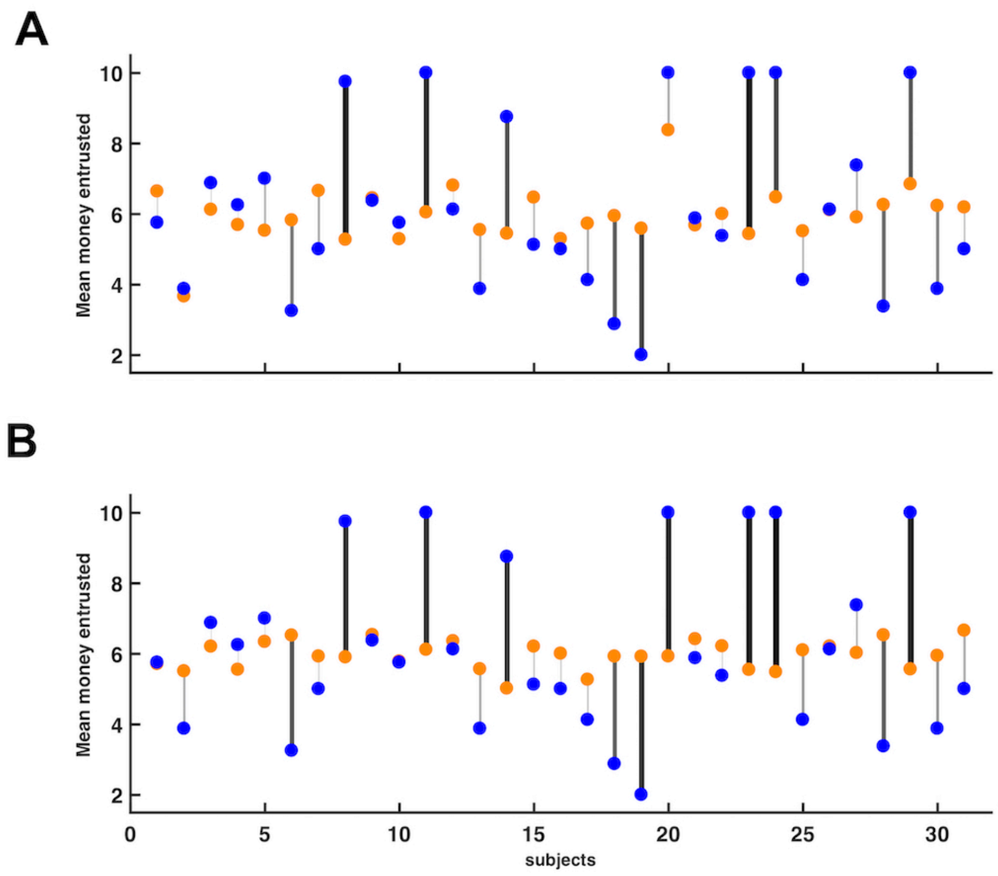

Supplementary Figure 4. Model-based predictions of economic trust decisions.

To test whether neural signatures of trustworthiness predict individual trusting behavior, multivariate regression analyses were performed with neural signal from the trustworthiness decoding network (**A**) and the value decoding network (**B**) to predict average individual trust in the trust game. Depicted in blue is the individual observed trust, in orange the predicted trust. Black lines connecting blue and orange dots represent model's prediction errors with thicker and darker lines reflecting bigger errors. Each dot represents a participant.

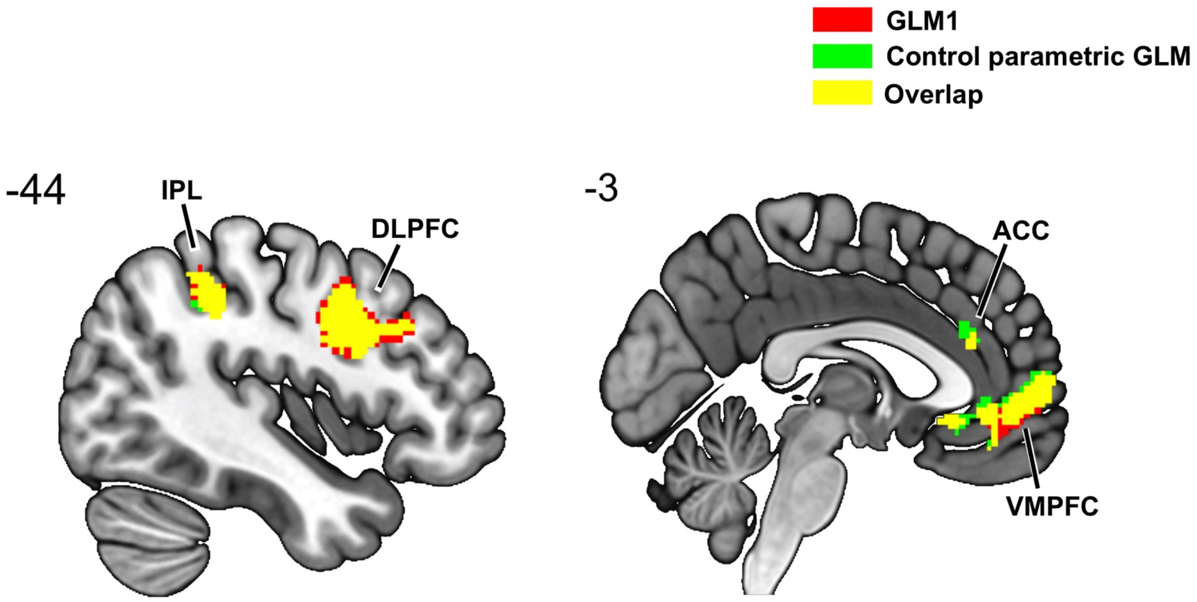

Supplementary Figure 5. Control parametric GLM analysis.

Comparison of univariate results based on GLM1 coding for honesty and dishonesty (red), and on the control parametric GLM with only one feedback regressor and a parametric modulator for honesty/dishonesty (green) orthogonalized to the parametric modulator for gain/loss. The two GLMs yielded similar results. In yellow are the overlaps depicted.
GLM, general linear model; IPL, inferior parietal lobule; DLPFC, dorsolateral prefrontal cortex; ACC, anterior cingulate cortex; VMPFC, ventromedial prefrontal cortex.

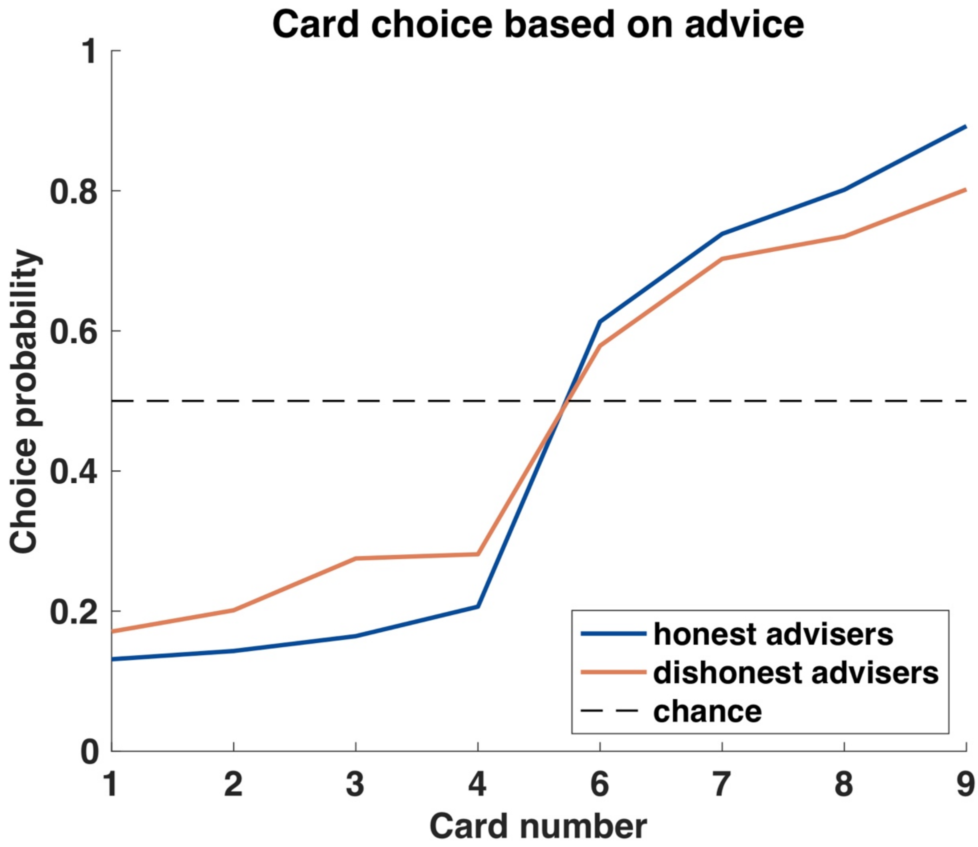

Supplementary Figure 6. Card-choice probability analysis.

Advice-taking behavior in the take advice game (TAG) was operationalized as the probability of choosing a card given the informativeness of the advice received. The optimal strategy in the game would be to choose more frequently a card when the adviser communicated that a number bigger than five is on that card but choose the other card when the adviser communicated that a number smaller than five is on that card. Moreover, as we manipulated the advisers' honesty, participants should have employed the optimal card-choice strategy differently for honest and dishonest advisers. In particular, they should have used this strategy more loosely for dishonest advisers compared to honest advisers, as they should discount the advice from the former. Analyses of card choice probabilities confirmed our hypotheses. Depicted is the card-choice probability for each advised card number across participants for honest and dishonest advisers.

Supplementary Tables

Supplementary Table 1. Multivariate results.

Hemisphere	Brain Region	Anatomical label	BA (PCM)	MNI Coordinates (mm)			*k*	T
				x	y	z		
**Trustworthiness decoding network**								
	PCC		BA 23	0	-52	34	498	5.59
**L**	IPS		BA 39 (hlP1)	-38	-56	48	858	5.05
**L**	DLPFC	IFG (p. Triangularis)	BA 44	-40	14	28	1502	4.95
**R**	DLPFC	middle frontal gyrus	BA 44	38	12	40	804	4.55
**Value decoding network**								
**R**	MCC		BA 32	14	38	28	22,111	10.19
**R**	IPL	supramarginal gyrus	BA 40 (PFm)	54	-44	30	1,688	8.42
**R**	Cerebellum	lobule VIIa	(Crus2, Hem)	40	-62	-42	626	8.05
**L**	SPL		BA 7 (7a)	-24	-60	62	994	7.91
**R**	Amygdala		(AStr)	32	-8	-12	62	7.05
**R**	FG		BA 37 (FG3)	36	-54	-10	57	6.94
**R**	STG		BA 22 (TE3/PF)	68	-38	10	80	6.89
**R**	PG		BA 2	42	-30	38	275	6.81
**R**	IPL		BA 40 (2/hlP3)	32	-44	52	197	6.74
**L**	PG		BA 2 (2)	-50	-32	52	478	6.70
**R**	STG		BA 22	60	-8	-10	107	6.67

			(TE3)					
**R**	Precuneus			4	-52	38	518	6.42
**R**	MCC		BA 23	2	-20	44	49	6.33
**R**	IPL	angular gyrus	BA 39 (PGa)	44	-64	54	32	6.08
**L**	IPL	middle occipital gyrus	BA 39 (PGp)	-42	-74	26	41	5.84

PCC, posterior cingulate cortex; IPS, intraparietal sulcus; DLPFC, dorsolateral prefrontal cortex; IFG, inferior frontal gyrus; MCC, middle cingulate cortex; IPL, inferior parietal lobule; SPL, superior parietal lobule; FG, fusiform gyrus; STG, superior temporal gyrus; PG, postcentral gyrus; R, right; L, left; BA, Broadman Area; PCM, probabilistic cytoarchitectonic map based on the SPM Anatomy toolbox.

Supplementary Table 2. Univariate contrasts for honesty and dishonesty.

Hemisphere	Brain Region	Anatomical label	BA (PCM)	MNI Coordinates (mm)			*k*	T
				x	y	z		
**Honesty > Dishonesty**								
	VMPFC	superior medial gyrus	BA 10 (Fp2)	0	56	0	637	4.69
**R**	ACC		BA 24	6	30	20	183	4.87
**Dishonesty > Honesty**								
**L**	DLPFC	precentral gyrus	BA 44	-40	2	30	1041	5.95
**L**	IPS		BA 7 (hIP3)	-30	-64	46	512	5.38
**L**	IPL		BA 40 (PFt)	-42	-38	40	287	4.95
**R**	DLPFC	IFG (p. Opercularis)	BA 44/45	46	14	32	275	4.30

VMPFC, ventromedial prefrontal cortex; ACC, anterior cingulate cortex; DLPFC, dorsolateral prefrontal cortex; IPS, intraparietal sulcus; IPL, inferior parietal lobule; IFG, inferior frontal gyrus; R, right; L, left; BA, Broadman Area; PCM, probabilistic cytoarchitectonic map based on the SPM Anatomy toolbox.

Supplementary Table 3. Univariate contrasts positive vs. negative feedback for honesty and dishonesty.

Hemisphere	Brain Region	Anatomical label	BA (PCM)	MNI Coordinates (mm)			*k*	T
				x	y	z		
**Honesty**								
**L**	striatum			-10	4	-8	3948	10.29
**L**	OFC	middle orbital gyrus	BA 11 (s32)	-8	44	-10	1089	6.90
**R**	precentral gyrus		BA 4 (4a)	32	-26	74	1190	5.60
**Dishonesty**								
**R**	striatum			14	8	-6	231	6.80

OFC, orbitofrontal cortex; R, right; L, left; BA, Broadman Area; PCM, probabilistic cytoarchitectonic map based on the SPM Anatomy toolbox.

Supplementary Table 4. Univariate contrasts negative vs. positive feedback for honesty and dishonesty.

Hemisphere	Brain Region	Anatomical label	BA (PCM)	MNI Coordinates (mm)			*k*	T
				x	y	z		
**Honesty**								
**R**	DLPFC	IFG (p. Opercularis)	BA 44	54	22	34	441	5.73
**R**	IPS		BA 7 (hIP3)	34	-54	50	503	5.40
**Dishonesty**								
**L**	IPL		BA 40 (hIP1)	-30	-50	40	424	5.46
**L**	MCC	posterior-medial frontal gyrus	BA 32	-8	12	48	328	5.33
**R**	DLPFC	IFG (p. Triangularis)	BA 44	40	16	28	402	4.92

DLPFC, dorsolateral prefrontal cortex; IFG, inferior frontal gyrus; IPS, intraparietal sulcus; IPL, inferior parietal lobule; MCC, middle cingulate cortex; R, right; L, left; BA, Broadman Area; PCM, probabilistic cytoarchitectonic map based on the SPM Anatomy toolbox.

Supplementary Table 5. Honesty and dishonesty parametric modulation of positive and negative feedback.

Hemisphere	Brain Region	Anatomical label	BA (PCM)	MNI Coordinates (mm)			*k*	T
				x	y	z		
**Honesty modulation of positive feedback**								
**L**	OFC	middle orbital gyrus	BA 11 (s32)	-4	42	-10	1626	5.34
**L**	OC	middle occipital gyrus	BA 19 (hOc4la)	-48	-76	2	182	4.99
**Dishonesty modulation of positive feedback**								
**R**	IPL		BA 40 (hlP3/hlP2/ PFm)	46	-48	52	386	4.58
**Honesty modulation of negative feedback**								
no suprathreshold voxels								
**Dishonesty modulation of negative feedback**								
**L**	Precentral gyrus		BA 6	-44	-2	24	395	5.54
**L**	IPS		BA 7 (hlP3)	-30	-62	48	689	4.96
**L**	IPL		BA 40 (PFt/hlP2)	-42	-38	40	172	4.40

OFC, orbitofrontal cortex; OC, occipital cortex; IPL, inferior parietal lobule; IPS, intraparietal sulcus; R, right; L, left; BA, Broadman Area; PCM, probabilistic cytoarchitectonic map based on the SPM Anatomy toolbox.
